# Zero-bias photocurrent in ferromagnetic topological insulator

**DOI:** 10.1038/ncomms12246

**Published:** 2016-07-20

**Authors:** N. Ogawa, R. Yoshimi, K. Yasuda, A. Tsukazaki, M. Kawasaki, Y. Tokura

**Affiliations:** 1 RIKEN Center for Emergent Matter Science (CEMS), Wako, Saitama 351-0198, Japan; 2 Department of Applied Physics and Quantum-Phase Electronics Center (QPEC), University of Tokyo, Tokyo 113-8656, Japan; 3 Institute for Materials Research, Tohoku University, Sendai 980-8577, Japan

## Abstract

Magnetic interactions in topological insulators cause essential modifications in the originally mass-less surface states. They offer a mass gap at the Dirac point and/or largely deform the energy dispersion, providing a new path towards exotic physics and applications to realize dissipation-less electronics. The nonequilibrium electron dynamics at these modified Dirac states unveil additional functions, such as highly efficient photon to spin-current conversion. Here we demonstrate the generation of large zero-bias photocurrent in magnetic topological insulator thin films on mid-infrared photoexcitation, pointing to the controllable band asymmetry in the momentum space. The photocurrent spectra with a maximal response to the intra-Dirac-band excitations can be a sensitive measure for the correlation between Dirac electrons and magnetic moments.

## Abstract (web summary)

By magnetic-doping, the electronic band structure of a topological insulator can be significantly altered to yield functional behaviour. Here, the authors demonstrate a large photocurrent response, and its control, under zero-bias in an optimally-designed magnetically-doped topological insulator thin film.

## Graphical abstract

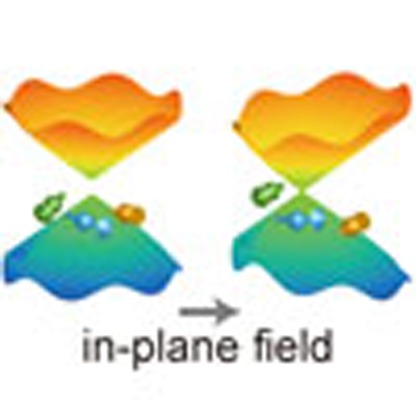

Bismuth-chalcogenides-based topological insulators^1^ (TIs) generally have the bulk band gap of several hundreds meV, where the conduction and valence bands are connected by the surface states: mass-less Dirac dispersions with spin-momentum locking. When doped with magnetic elements^2^, a Dirac-mass gap opens and/or the energy dispersion deforms (Fig. 1a) through the breaking of time-reversal symmetry^3^,^4^,^5^. The toplogical character of the surface states survives up to a moderate doping density, and offer a fertile ground of quantum phenomena, for example, creation of magnetic monopoles^6^, quantized magnetoelectric effects^7^,^8^,^9^,^10^,^11^,^12^ and quantum anomalous Hall effects^13^,^14^,^15^.

The corresponding Hamiltonian can be expressed as^16^


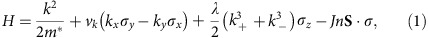


where each term represents particle-hole asymmetry (*k* is momentum, and m* the effective mass), spin-orbit interaction resulting in the helical spin state (*v*_*k*_ is velocity and σ the Pauli matrices), hexagonal warping with amplitude *λ* (*k*_±_≡*k*_*x*_±*ik*_*y*_), and coupling of electron's spin to localized dopant spin **S** (*n* is the density and *J* is the interaction energy), respectively^5^,^16^,^17^, with the unit of *ħ*=1. Here we take Cartesian coordinates with *z* along the surface normal. For the out-of-plane alignment of the dopant moments, either by magnetic anisotropy or by external fields, the spins turn parallel to *z* near the gapped Dirac point in the energy spectrum, and the Dirac-mass gap scales with *JnS*_*z*_. This dispersion shows 3-fold symmetry around the *k*_*z*_ axis^5^, leading to an isotropic optical response in the linear regime. The Dirac-mass gap 2Δ of 60 meV was experimentally confirmed recently on a local scale in the case of Cr-doped (Bi,Sb)_2_Te_3_ ferromagnetic TIs^18^. For the case of in-plane magnetization, for example, the finite *S*_*y*_, the gap closes or substantially reduces, with the Dirac point shifted from the zone center along *k*_*x*_ due to the Zeeman effect (Fig. 1a). Accordingly, the constant energy contour deforms by leaving one mirror plane in the momentum space^5^. This asymmetry can induce transient spin and charge currents on photoexcitation^19^.

In addition to intensive optical spectroscopy^20^,^21^,^22^,^23^,^24^,^25^,^26^,^27^, a number of photoeffects in TIs have been studied both theoretically and experimentally; photocurrent generation and galvanic effects^28^,^29^,^30^,^31^,^32^, carrier/spin dynamics elaborated by time-resolved optics^33^,^34^,^35^,^36^,^37^,^38^,^39^,^40^,^41^, photoemission^42^,^43^,^44^,^45^,^46^,^47^,^48^,^49^,^50^,^51^ and the Floquet state^52^, including the cases of thin films with magnetic dopants^53^,^54^, to name a few. For the photocurrent generation at the normal incidence, it is predicted that the orbital coupling of light, that is, *ħ*
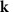
→*ħ*
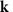
−*e*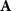, provides a predominant contribution to the polarization-independent photocurrent under an in-plane external magnetic field^17^.

In this paper, we study photocurrent spectra in Cr_0.3_(Bi_0.22_Sb_0.78_)_1.7_Te_3_ (CBST) thin films with a thickness of 8 nm. These samples are designed to achieve the following properties: The Fermi level is tuned near the Dirac point, which is isolated from the bulk states^18^,^55^. The top and bottom surfaces hosting Dirac states are decoupled with the minimal contribution of the bulk volume in between. The dopant concentration is increased from those of the previous works^13^,^14^,^15^ to enhance magnetic transition temperature, *T*_C_. Thus, intrinsic signals from the Dirac electrons^49^ interacting with magnetic dopants can be explored at moderate temperature. We show the generation of large zero-bias photocurrent on mid-infrared photoexcitation at normal incidence (Fig. 1b), realized by the field-controllable band asymmetry in the momentum space. The enhanced response at the intra-Dirac-band excitation would reveal intrinsic interactions between Dirac electrons and magnetic moments.

## Results

### Transport properties

**Figure 1:**
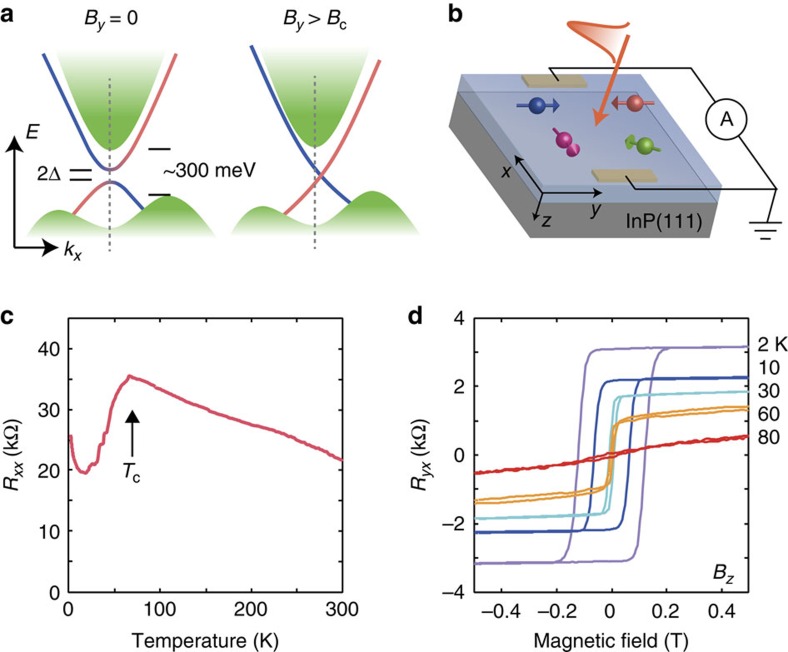
Schematic energy dispersion and transport properties. (**a**) Electronic structure of magnetic topological insulator: the mass gap 2Δ at the Dirac point, and the effects of in-plane magnetic field above the critical value *B*_*c*_ inducing finite in-plane portion of the dopant spin *S*_*y*_. (**b**) Experimental setup with the coordinates used in this work. The film is illuminated at normal incidence, and the photocurrent along *x* direction is measured. (**c**) Longitudinal and (**d**) Hall resistance of the Cr_0.3_(Bi_0.22_Sb_0.78_)_1.7_Te_3_/InP(111) film, indicating the magnetic transition temperature *T*_C_ and ferromagnetic response of the surface Dirac states with uniaxial anisotropy.

Figure 1c shows that the fabricated CBST film is non-metallic at room temperature, and the conduction through the surface states becomes apparent slightly below the *T*_C_∼75 K, due to the freezing of thermally excited carriers in the bulk state and also reduced magnetic impurity scatterings^14^ (Supplementary Fig. 1). The Hall resistivity data (Fig. 1d) also illustrate the *T*_C_ and clear magnetic hysteresis loops at lower temperatures. In fact, the thin-film sample, prepared at the similar growth condition but with smaller Cr concentration, was proven to show the quantum anomalous Hall effect as the hallmark of the above features of our sample design^14^.

### Zero-bias photocurrent spectra

**Figure 2:**
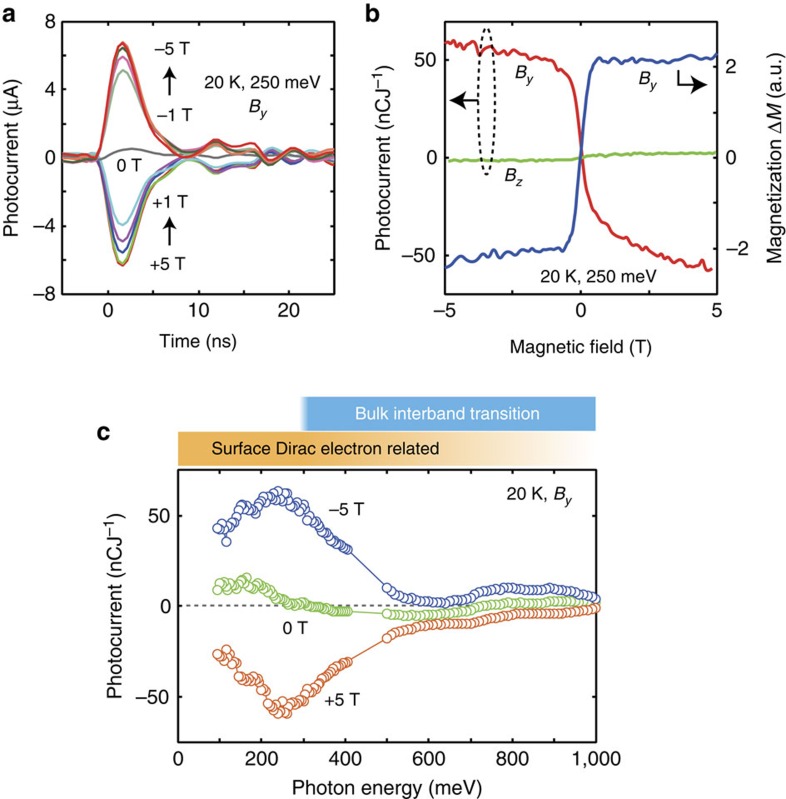
Photocurrent characteristics of the magnetic topological insulator thin film. (**a**) Zero-bias photocurrent at 250 meV (and 350 nJ) excitation under varying in-plane magnetic field *B*_*y*_. (**b**) Normalized photocurrent under in-plane (*B*_*y*_) and out-of-plane (*B*_*z*_) magnetic fields, plotted together with the magnetization under *B*_*y*_. (**c**) Photocurrent spectra at 20 K under *B*_*y*_.

Figure 2a,b show the representative zero-bias photocurrent under the in-plane (*B*_*y*_) and out-of-plane (*B*_*z*_) external magnetic fields measured at 20 K, compared with the sample magnetization (Δ*M*, after subtracting the substrate contributions). Only in the case of applying *B*_*y*_, a large photocurrent was detected. The current is nearly proportional to the in-plane magnetization; it reverses when the magnetic-field direction (or spin *S*_*y*_) is flipped. A slight asymmetry at the positive and negative fields could be ascribed to the nonequivalent electrodes geometry. The photocurrent spectra show a pronounced peak around 250 meV (Fig. 2c). These characteristics were found to be independent of the incident photon polarization^17^. The bulk band gap is anticipated to be around 300 meV, therefore the low-energy photoresponses, at least <300 meV, can be ascribed to the dynamics of the spin-polarized Dirac electron at the surface. There exist some fluctuations in the photocurrent spectra, such as the increase of photocurrent around 100 meV under 0 T, whose origins are not clear at this stage.

### Temperature and magnetic-field dependence

**Figure 3:**
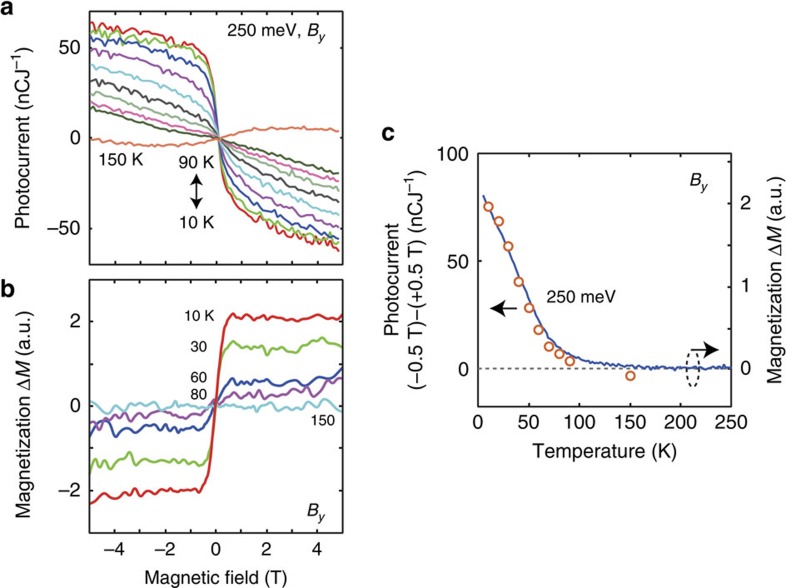
Temperature dependence of the zero-bias photocurrent. (**a**) Normalized zero-bias photocurrent under in-plane magnetic field *B*_*y*_ at varying temperature. (**b**) Magnetization under *B*_*y*_. (**c**) Photocurrent at ±0.5 T as a function of temperature, plotted together with magnetization.

Detailed characteristics of the zero-bias photocurrent are shown in Fig. 3. The inflection behaviour in the magnetic-field dependence diminishes around 80 K (Fig. 3a), consistent with the trend of in-plane magnetization (Fig. 3b) and the Hall resistance data (Fig. 1d). The temperature dependence of the photocurrent nicely follows that of the magnetization (Fig. 3c).

## Discussion

For the case of normal incidence in general, an imbalance in the electron excitation at the opposite *k*, and nonequilibrium carrier distributions on relaxation, can trigger the generation of directional photocurrent^19^. This photocurrent can be enhanced by the spin-orbit interaction, most prototypically for the surface Dirac electrons with the deformed Dirac dispersions as in the present case. In our experiments, possible contributions from the photogalvanic effect due to the trigonal warping can be neglected, judging from the absence of photocurrent at zero field. Neither the Nernst-Ettingshausen effect nor the photon drags have roles in our optical geometry.

**Figure 4:**
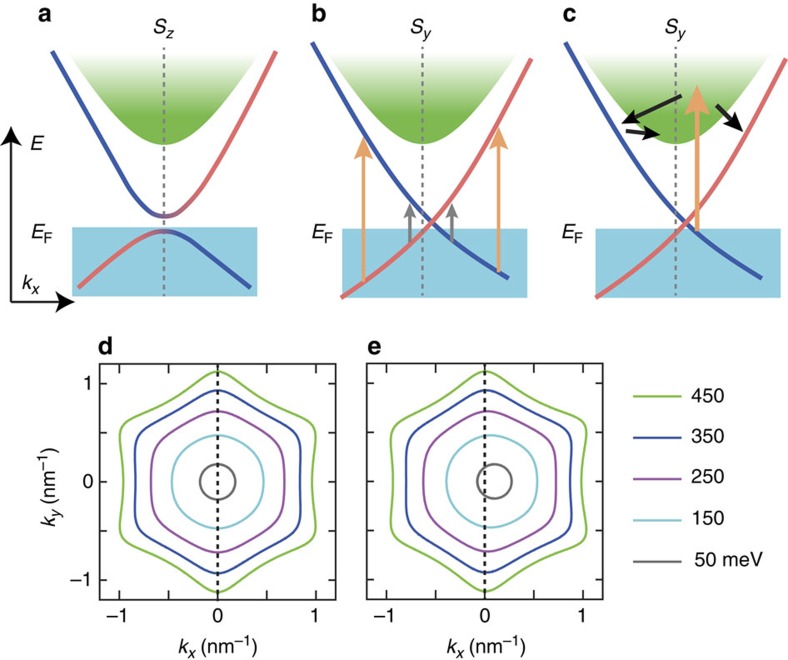
Mechanism of zero-bias photocurrent generation. Schematics for the energy dispersion in the magnetic topological insulator for the out-of-plane *S*_*z*_ (**a**) and in-plane *S*_*y*_ (**b**,**c**) alignment of dopant spin. Corresponding energy contours calculated from equation (1) are depicted in (**d**,**e**). Possible imbalance in the photoexcitation at the opposite *k*_*x*_ is shown in **b**: grey and orange arrows for low- and high-photon-energy excitations, respectively. The scattering between the surface and bulk states is shown in **c**.

The observed zero-bias photocurrent in the magnetic TI thin film can be explained by the magnetization-induced modification of the energy dispersion (Fig. 4), making both the excitation and relaxation at ±*k* asymmetric. In the absence of external fields, or under the application of *B*_*z*_, the doped Cr moment induces the Dirac-mass gap. In this case, the constant energy contour of the dispersion remains symmetric in the *k*_*x*_-*k*_*y*_ plane (Fig. 4a,d). In stark contrast, with the application of −*B*_*y*_ (induction of *S*_*y*_), the Dirac-mass gap closes, and the energy contour shifts/deforms orthogonal to the orientation of magnetic moment (towards *k*_*x*_ for *S*_*y*_) (Fig. 4b,e). Here we draw the energy contours in Fig. 4d,e, by following equation (1) with the parameters in refs 5, 19, and 2Δ=60 meV from ref. 18. These shifts and deformations are proportional to the in-plane moment *S*_*y*_, and the latter have a major role for the photocurrent generation.

When the photon energy is below 300 meV, within the bulk band gap where the observed photocurrent largely enhances, the photoexcitation between the bulk valence and conduction bands can be neglected. Given that the Fermi level is located near the Dirac point, we can expect two pathways of Dirac electron-related excitation and relaxation; excitation within the surface states (Fig. 4b), and between the surface states and the bulk states (and vice versa) (Fig. 4c). We will exclude the latter process as the major mechanism of photocurrent generation in the following, since merely small photocurrent observed at the higher photon energy (Fig. 2c). The surface state as the final state of the optical excitation is important for the observed photocurrent, where the asymmetry in the dispersion is directly involved.

Now we discuss the spectral structure as exemplified by the peak around 250 meV (Fig. 2c). The optical processes possibly involved in the present photogalvanic effect have been discussed theoretically in refs 5, 19. In short, the zero-bias photocurrent flows due to the unbalanced transient carrier population (*f*(*k*): Fermi-Dirac function) in the deformed Dirac dispersions, that is, *f*(*k*_*x*_)−*f*(−*k*_*x*_), multiplied by the group velocity, which can be proportional to the local magnetic moment. The optical absorption in the surface Dirac states is supposed to be nearly monotonic as a function of photon energy in our spectral range^19^,^21^,^22^,^23^,^24^,^25^,^54^ (see also Supplementary Fig. 2). However, the observed photocurrent shows a clear peak around 250 meV; it decreases towards zero in lowering photon energy and also shows a reduction in increasing photon energy above 250 meV. The former trend is predicted in ref. 19 as due to the decreased asymmetry near the Dirac point (compare the grey and orange transitions in Fig. 4b). Even with the finite optical absorption, the photocarriers with the opposite group velocity would cancel for the low-energy excitation. The decrease in the photocurrent at higher energy can be ascribed to the pronounced scattering between bulk and surface states (Fig. 4c), which is not taken into account in ref. 19. Here the optical transition to the bulk states increases in the photon energy region above ∼250 meV, and most of the population asymmetry is lost above 500 meV due to the scattering between the bulk and surface states. Note that we have finite potential fluctuations in the film, including those of ∼20 meV from Cr dopants^18^ and also between the two surface states (see below), which can induce broadenings in the photocurrent spectra. The small but finite photocurrent under *B*_*z*_ (Fig. 2b) may point to the effect of hexagonal warping with the spin moments oriented along *z*, which is ignored in the above analyses^5^.

Considering the thickness of our sample (8 nm), the top and the bottom surface states (the latter is the interface between CBST and InP) experience nearly the same optical fields for the low-photon-energy region (Supplementary Fig. 2). In the above-discussed model, the energy contour shifts/deforms in the opposite direction for the top and bottom surface states; the excited photocarriers flow in the counter direction, which may cancel at the end. However, in general, the top and bottom surface states are inevitably inequivalent^9^ due to their distinct environments and growth characteristics. The energy shift of the surface-state electronic bands is estimated as large as 50-70 meV from the quantum Hall effect measurements^56^. Thus we detect the photocurrent portion not cancelled in the two surface states. By checking the signals from the sample with a modulated Cr doping, we found an indication that the bottom surface has the larger contribution to the photocurrent in the present case (Supplementary Fig. 3). It is also seen that we can possibly enhance the photocurrent by further differentiating the top and bottom surface states. However, a quantitative discussion is difficult at this stage because of the extrinsic factors such as the change in the transition temperature and scatterings by dopants.

The amplitude of the detected photocurrent, although transient, reaches a value as large as 60 μA cm^−1^ (Fig. 2a with the experimental geometry taken into account). This is more than two orders of magnitude larger than that observed by the circular photogalvanic effect in the bulk Bi_2_Se_3_ (ref. 30), in which the oblique-incidence circularly polarized light can inject the spins in the Dirac dispersion to generate the zero-bias current. This is also larger than the theoretically predicted values with magnetic proximity interactions^19^ (see also Supplementary Fig. 4). Considering the relaxation of photoexcited carriers before reaching the electrodes, the instantaneous photocurrent would be even much larger. In the experiments shown above, we used the external magnetic field larger than ∼0.5 T to orient the Cr moments (and to generate the photocurrent). If we could prepare the sample with an easy-plane anisotropy, it would be possible to have a spin-polarized photocurrent in zero field, whose direction could be controlled by the switching of the magnetic domains. As such, the magnetic control of the nonequilibrium Dirac electrons in the magnetic topological insulator will pave a new path for intriguing spintronic functions.

## Methods

### Sample preparation and characterization

Thin films of CBST were grown on InP(111) substrates by the molecular beam epitaxy, where the similar films with the smaller Cr concentration have been proven to show the quantum anomalous Hall effect. The detailed growth conditions are described in ref. 14. The surface was protected by a 3 nm thick Al_2_O_3_ cap layer. The samples were characterized by conventional transport and magnetization measurements. The diamagnetic contribution of the substrate was subtracted for the latter.

### Photocurrent detection

Two AuPd electrodes were deposited along the [10
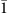
0] axis after removing the cap layer. The short-circuit photocurrent was measured through a wide-band preamplifier (bandwidth of 200 MHz) with the illumination by a pulsed laser source (120 fs, 1 kHz, spot size 1.0–0.5 mm in diameter) at normal incidence. The excitation spot was carefully positioned at the center of the sample to cancel possible thermoelectric signals (Supplementary Fig. 5). The time trace of the photocurrent was averaged 150 times in the digitizing oscilloscope, which was broadened by the response of the preamplifier and followed by ringing due to the impedance mismatch in the circuit (Fig. 2a). The magnetic field and photon energy dependencies were obtained by averaging more than 2,048 signal pulses after gating the photocurrent signal by a boxcar averager. The acquired signal was normalized by the integrated time-trace from the oscilloscope, as a number of charge, and also by the incident photon intensity. The error bars in our measurements are within the circle marks in Fig. 2c. We have checked three samples with slightly different compositions, and confirmed nearly the identical results.

### Data availability

The data that support the findings of this study are available from the corresponding author on request.

## Additional information

**How to cite this article:** Ogawa, N. *et al*. Zero-bias photocurrent in ferromagnetic topological insulator. *Nat. Commun.* 7:12246 doi: 10.1038/ncomms12246 (2016).

## Supplementary Material

Supplementary Information Supplementary Figures 1-5 and Supplementary References
