# Supplementary material for: Zero-bias photocurrent in ferromagnetic topological insulator
Source: Nat Commun. 2016 Jul 20;7:12246. doi: 10.1038/ncomms12246 (PMC4961789; doi:10.1038/ncomms12246)
Supplement: Supplementary Information — Supplementary Figures 1-5 and Supplementary References [file ncomms12246-s1.pdf]

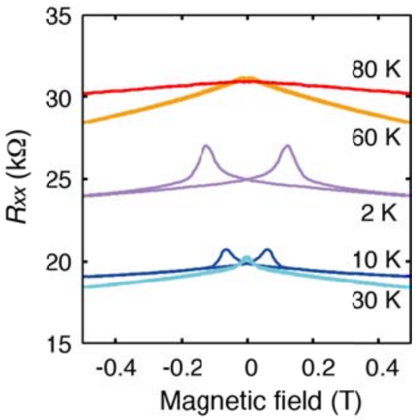

Supplementary Figure 1. **Longitudinal resistance as a function of out-of-plane magnetic field.** The $Cr_{0.3}(Bi_{0.22}Sb_{0.78})_{1.7}Te_3$ (CBST) thin film (t = 8 nm) on a InP(111) substrate shows negative magnetoresistance. The electron transport illustrated in Fig. 1c in the main text is a result of intricate competition between the thermal excitation, impurity and surface/interface scatterings, and formation of a mass gap. Through the alignment of the local moments, the scattering of the conduction electron decreases, leading to the reduction in the resistance at T_C. The real insulating character from the mass gap appears only at the lowest temperature.

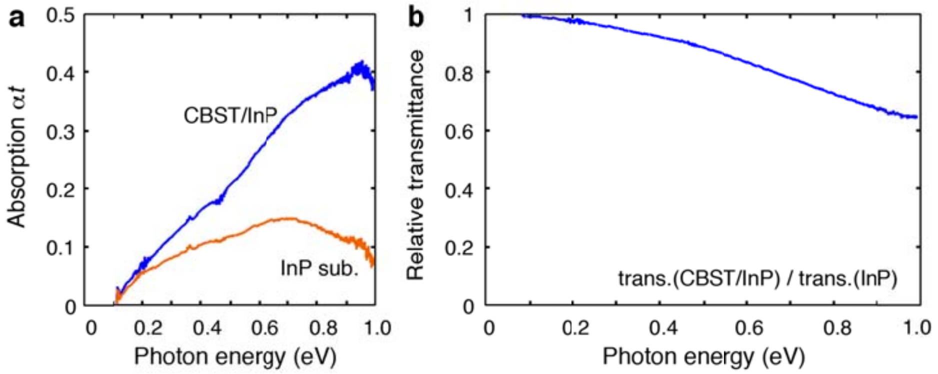

Supplementary Figure 2. **Optical characteristics of the CBST film.** (a) Absorption and (b) transmittance of a CBST film at room temperature. The relative transmittance is deduced by comparing the optical transmission with and without the CBST film deposited on the InP substrate. The samples are

capped with the same thickness of Al_2O_3 for protection. It is seen that the transmittance decreases monotonically with increasing photon energy, consistent with previous reports[1], although the bulk band gap of the CBST is not clearly discernible.

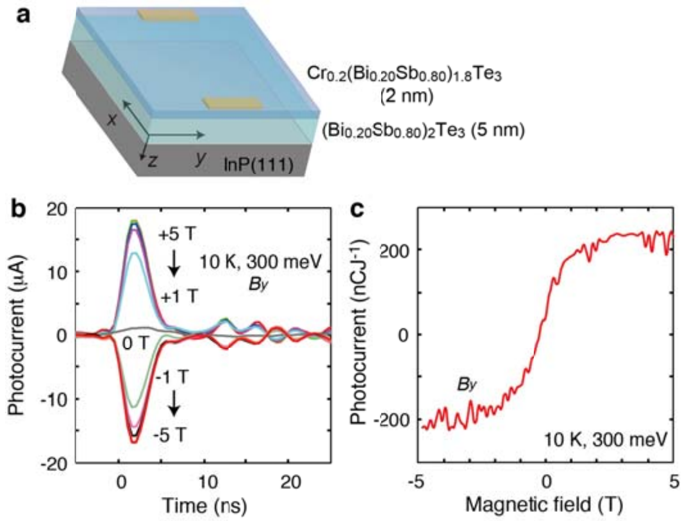

Supplementary Figure 3: **Photocurrent in a CBST film with modulated Cr doping.** For the generation of zero-bias photocurrent in our ferromagnetic topological insulator thin films (Fig. 4 in the main text), it is expected that the photocarriers excited at the top and bottom surface states would cancel by flowing in the opposite direction. However, in reality, the electronic states at these surfaces are not equivalent, as discussed in the main text. Therefore the uncanceled portion of the current is always anticipated and in fact detected, leaving some possibilities of even enhancing the total photocurrent. (a) Schematic illustration of $Cr_{0.2}(Bi_{0.20}Sb_{0.80})_{1.8}Te_3$(2 nm)/$(Bi_{0.20}Sb_{0.80})_2Te_3$(5 nm) bilayer sample. For this film, the external magnetic field predominantly modulates the Dirac dispersions at the top surface (the Al_2O_3/CBST interface). (b) Zero-bias photocurrent at 300 meV (200 nJ) excitation under varying in-plane (B_y) magnetic field from +5 to −5 T. (c) Magnetic-field dependence of photocurrent. In (b) and (c), clear differences can be seen from those in the

homogeneously doped CBST film (main text): The current flows in the opposite direction, and with nearly 3 times higher generation efficiency. The former indicates that the bottom surface generates slightly larger photocurrent for the case of the single layer CBST film, and the latter manifests that we can largely enhance the photocurrent by lifting the equivalence between the top and bottom surface states.

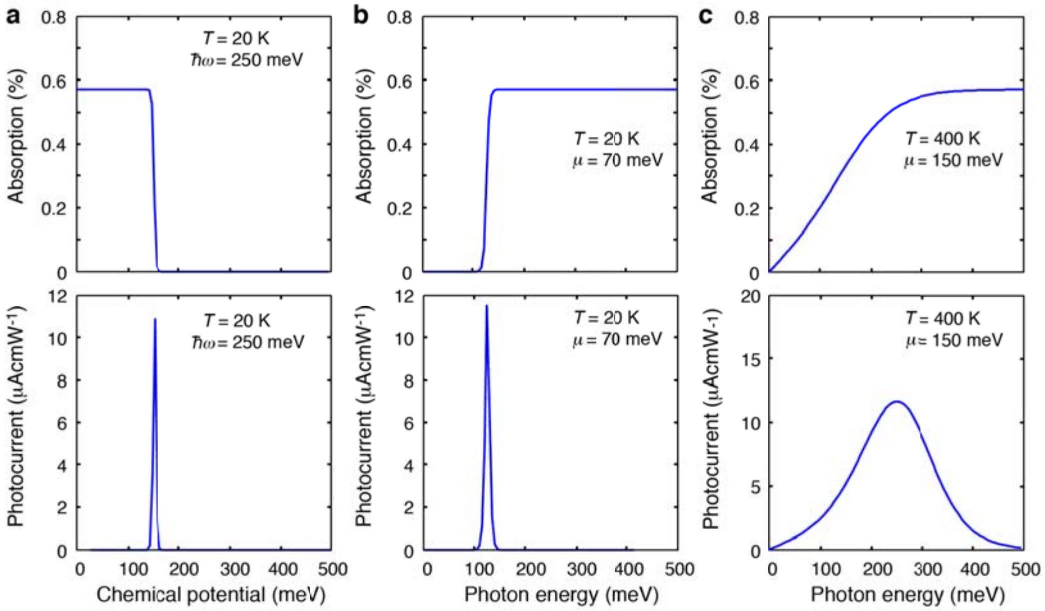

Supplementary Figure 4. **Comparison with theoretical predictions.** Based on ref. 2 (the photogalvanic effect in TIs with magnetic proximity interactions), the simulated optical absorption and photocurrent are plotted by replacing some of the parameters to those from our experimental values. (a) Variation of chemical potential at the incident photon energy of 250 meV, corresponding to the energy at the maximum photoresponse in the experiment. (b) Variation of photon energy with the chemical potential assumed at 70 meV, which is at the maximum of estimated energy shift between the surface-state electronic bands. (In reality, the Dirac point locates inside this energy region, therefore the chemical potential will be much smaller.) Other parameters used are;

temperature of 20 K, Fermi velocity v_F = 4.28×10^7 cms^{-1}, coefficient of the quadratic dispersion D =13 eVÅ^2, and exchange energy G = 40 meV. The latter predominantly scales the amplitude of photocurrent and does not affect the spectral shape. It is seen that a narrow photocurrent peak is now expected around 120 meV (a). (c) Temperature and the chemical potential are tuned to reproduce the experimentally-observed photocurrent peak. As has been discussed in the Fig. 3(c) in ref. 2, the chemical potential of 150 meV is necessary to explain the photocurrent peak emerging at 250 meV, which is more than two times larger than our experimental estimation. In addition, the temperature of 400 K is needed to mimic the observed broadening in the spectrum. Judging from the close resemblance between the zero-bias photocurrent and magnetization in the temperature scan (Fig. 3c in the main text), it is expected that the quasi-equilibrium sample temperature is not substantially increased upon photoexcitation. Consideration of trigonal warpings[3], potential fluctuations, transient electron temperature upon sub-picosecond laser excitation, and transitions between surface and bulk states would help improve the discrepancies between the simulation and experiments.

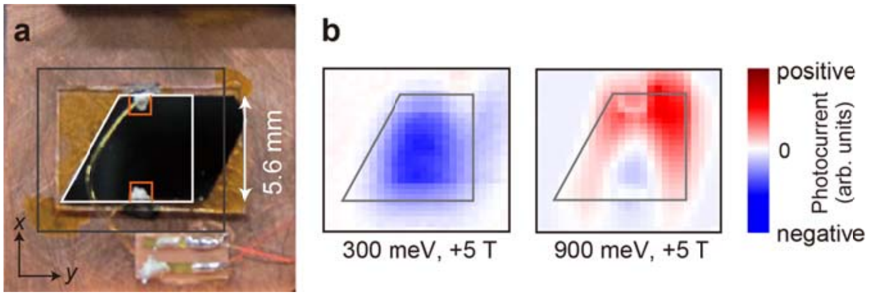

Supplementary Figure 5. **Locating the laser spot.** To evaluate/exclude extrinsic signals in the photocurrent, such as those from thermoelectric effects, the excitation spot was scanned to map out the signal prior to each measurement.

(a) Photograph of the sample. A white trapezoid indicates the area where the CBST film is deposited, and orange squares outline the AuPd electrodes. The black square shows the region where the excitation laser spot is raster-scanned. (b) Photocurrent mappings measured at 20 K under B_y = +5 T. The gray trapezoid corresponds to that in (a) (with white color). Note that the AuPd electrodes are barely visible. At low photon energy (300 meV), corresponding to the region of enhanced photoresponse discussed in the main text, the photocurrent appears almost homogeneously across the film surface. At higher photon energy (900 meV), on the contrary, the photocurrent changes its sign along the line connecting the two electrodes, as has been observed in previous works[4]. The effect of magnetic field was found to be small for this high-energy signal. For the experiments in the main text, the laser spot was set at the location where this high-energy response nearly diminishes, which is usually at the center between the two electrodes. Note that these photocurrent images are blurred by the excitation spot size and also by the small fluctuation in the laser pointing. The photocurrent amplitudes are not directly comparable due to the adjustment of image contrast.

Supplementary References

1. Onishi, Y. et al. Ultrafast carrier relaxation through Auger recombination in the topological insulator $Bi_{1.5}Sb_{0.5}Te_{1.7}Se_{1.3}$. Phys. Rev. B **91,** 085306 (2015).

2. Semenov, Y. G., Li, X. & Kim, K. W. Tunable photogalvanic effect on topological insulator surfaces via proximity interactions. Phys. Rev. B **86,** 201401(R) (2012).

3. Henk, J. et al. Topological Character and Magnetism of the Dirac State in Mn-Doped Bi_2Te_3. Phys. Rev. Lett. **109,** 076801 (2012).

4. McIver, J. W., Hsieh, D., Steinberg, H., Jarillo-Herrero, P. & Gedik, N. Control over topological insulator photocurrents with light polarization. Nature Nanotech. **7,** 96-100 (2012).
